# A Multicomponent eHealth Intervention for Family Carers for People Affected by Psychosis: A Coproduced Design and Build Study

**DOI:** 10.2196/14374

**Published:** 2019-08-06

**Authors:** Jacqueline Sin, Claire Henderson, Luke A Woodham, Aurora Sesé Hernández, Steve Gillard

**Affiliations:** 1 Population Health Research Institute St George's, University of London London United Kingdom; 2 School of Psychology and Clinical Language Sciences University of Reading Reading United Kingdom; 3 Health Service and Population Research Department Institute of Psychiatry, Psychology and Neuroscience King's College London London United Kingdom; 4 Institute of Medical and Biomedical Education St George's, University of London London United Kingdom

**Keywords:** eHealth, family caregivers, psychosis, mental health, participatory research, public and patient involvement, coproduction

## Abstract

**Background:**

Psychosis, including schizophrenia, is the most common severe mental illness affecting 1% of the population worldwide. A large number of people provide long-term support and care for a relative with psychosis. Although psychoeducational interventions, especially those delivered through a face-to-face group format, have an established evidence base for improving the caregiving experience, well-being, and health outcomes, large-scale implementation and access remain limited. There is a demand for such provision to be made through the internet for greater flexibility and wider access.

**Objective:**

This study aimed to integrate participatory research methodologies by the public, patients, and carers into the eHealth (electronic health) intervention design and build process to improve the product’s usability and acceptability.

**Methods:**

We adapted a structured eHealth intervention build method to include participatory research activities involving key stakeholders and end users to co-design and coproduce our intervention. An expert advisory group (EAG) comprising public involvement members led the formative design and build work using an agile build process. Carers independent from the study were consulted on the evolving drafts of the intervention prototype through focus group meetings. These results were fed back into the intervention build work continuously to ensure end users’ input inform every stage of the process.

**Results:**

An EAG comprising individuals with lived experience of psychosis, carers, health care professionals, researchers, voluntary organization workers, and eLearning experts (n=14) was established. A total of 4 coproduction workshops were held over 1 year during which the alpha and beta prototypes were designed and built through the participatory research work. Alongside this, 2 rounds of focus group study with carers (n=24, in 4 groups) were conducted to seek consultation on end users’ views and ideas to optimize the intervention design and usability. Finally, the EAG carried out a Web-based walk-through exercise on the intervention prototype and further refined it to make it ready for an online usability test. The final product contains multiple sections providing information on psychosis and related caregiving topics and interactive discussion forums with experts and peers for psychosocial support. It provides psychoeducation and psychosocial support for carers through the internet, promoting flexible access and individualized choices of information and support.

**Conclusions:**

The participatory research work led to the coproduction of a eHealth intervention called COPe-support (Carers fOr People with Psychosis e-support). We believe the study methodology, results, and output have optimized the intervention design and usability, fitting the end users’ needs and usage pattern. COPe-support is currently being tested for its effectiveness in promoting carers’ health outcome through an online randomized controlled trial.

**Trial Registration:**

ISRCTN Registry ISRCTN89563420; http://www.isrctn.com/ISRCTN89563420

## Introduction

### Family Caregiving

With ever-advancing health care technologies and growing longevity worldwide, a significant proportion of people provide substantial and sustained help and support to friends or family members suffering from a long-term illness [1]. In the United Kingdom and the United States, nearly one-fourth of the adult population identifies itself as a carer for a loved one who is ill, disabled, or elderly [2,3]. Many of these carers support a loved one affected by a severe and long-term mental illness such as psychosis [4,5]. Family caregiving often covers a huge amount of care and support ranging from emotional and psychosocial support (eg, engaging their loved one in social activities and sharing ups and downs) to financial and practical support (eg, provision of financial and practical help and monitoring of health and treatment compliance). Compared with paid or professional care workers, family carers also have unique advantages in knowing the individual’s strengths and interests in addition to their needs. Most have a well-established emotional bond and are committed to use all these preexisting knowledge and relationships to support their loved one in their recovery [6-8]. Caregiving imparts paramount emotional and psychosocial benefits to the cared-for individuals such that people in receipt of support from their family or social network have better prognosis, fewer relapses, and higher quality of life compared with those without such support [9-12]. Collectively, caregiving by family members amounts to significant economic savings to the wider society [4,9].

Conversely, it is well established that caring demands can jeopardize carers’ well-being [2,13,14]. For instance, population-level research data repeatedly show that carers experience higher levels of distress and poorer well-being when compared with age-matched counterparts in the general population [10,15,16]. Indeed, distress in carers frequently reaches clinical thresholds, and their psychiatric symptom scores (eg, depression and anxiety) are found to be consistently inversely associated with the amount of care they provide, that is, carers’ mental health worsens with increasing demands on caregiving [2,10,15]. Research evidence also suggests that poor well-being may hamper carers’ caregiving capacity. Carers who feel they are not supported and lack resources (eg, information about the illness condition and related management issues) to cope are less likely to engage in caring for their loved ones or more likely to exhibit critical or hostile behavior toward the cared-for individuals, albeit unintentionally [13,14,17]. This, in turn, can impact negatively on both the carers and the cared-for individuals, leading to a vicious cycle of poor health and quality of life for all concerned.

### Psychosocial Interventions Targeting Carers

Consequently, a body of research has been undertaken to explore interventions, which can best support carers [14,18,19]. Among a range of psychosocial interventions targeting whole families and/or carers alongside the individual treatment regime, psychoeducation (ie, information giving on the illness condition and related caregiving and problem-solving strategies) has the strongest evidence base for its effectiveness in enhancing carers’ knowledge and coping with their caring roles [5,20]. Commonly based on the stress-appraisal-coping theory as applied in family caregiving [14,21-23], it has been hypothesized that psychoeducation, with education as its core feature and prime aim, works directly to improve carers’ knowledge about psychosis and related caregiving issues. Improved knowledge about coping strategies and available resources can lead to a more positive appraisal of their caregiving experiences and carers’ perceived self-efficacy in coping with the demands [14,24]. These, in turn, can translate into a more supportive home environment for all and better prognosis and reduced relapses in the cared-for individuals. In addition to information and advice on psychosis and related caregiving strategies, carers also identify that sharing mutual support and learning with other carers (ie, peers) as particularly useful in reducing their sense of isolation [18,20]. Consequently, psychoeducational interventions, especially those delivered in a group format, as a discrete treatment on their own or augmenting other treatments (eg, family intervention or mutual support programs) are widely recommended and practiced around the world [14,18,20].

### Carer-Specific eHealth Interventions

Carers have expressed their desire for interventions to support them to be delivered to them through a digital medium to fit with their caregiving and other commitments [25-27]. The internet offers the potential to deliver interventions, which are highly flexible, accessible, and yet adaptable to individualized needs and schedule. With the popularity of eHealth (electronic health; ie, health care practice delivered through the internet) and mobile Health (ie, through the mobile network) interventions targeting a wide range of common public mental health issues (eg, insomnia and stress management) growing fast, internet-based interventions targeting carers have gathered momentum and popularity in the recent decade [11,19,28]. eHealth interventions using an enriched online environment can integrate multiple components, especially educational and therapeutic information and network support with health care professionals and peers, and deliver such provisions to a critical mass of carers [11,29]. Carers, as the end users, particularly appreciate the autonomy that eHealth interventions offer, as they can decide which components or strategies resonate with them, how much time to spend accessing the intervention, and when to do so [28,30]. Systematic reviews on interventions targeting carers identify that eHealth interventions are particularly widespread in the field of dementia, stroke, or cardiovascular diseases. More importantly, research evidence to date shows promising effectiveness results in improving carers’ health outcomes [11,31]. Furthermore, these reviews suggest that effective Web-based interventions commonly have 3 essential ingredients. These include (1) multiple components such as information and well-being promotion strategies (including mindfulness and cognitive behavioral–oriented exercises), (2) psychoeducational content to enhance understanding of the illness condition and related caregiving issues, and (3) flexibility to self-pace and self-tailor the intervention to individual needs [11,19,31].

### Carers for People With Psychosis

Notwithstanding the evidence base for eHealth interventions targeting carers and their expressed desire for such provision, development in the field of psychosis appears to be lagging with few empirical studies documented to date. Of these studies, 2 were conducted in the United States nearly a decade ago. Rotondi et al developed and evaluated an online psychoeducational intervention with a peer forum for individuals with schizophrenia and their carers [32], and Glynn et al developed an online multifamily group program for carers of people with schizophrenia providing synchronous and asynchronous group sessions [33]. More recently, 1 study was conducted in the United Kingdom, trialing a fully Web-based psychoeducation and peer-support intervention for siblings of individuals who developed first episode psychosis (The E Sibling Project) [34]. A further psychoeducational intervention using an enriched online environment was developed and tested for acceptability in Hong Kong, China, targeting carers of people with psychosis [35]. All these interventions were highly valued by the carers, albeit in either a pilot trial or usability study with a relatively small sample size (n=21, 26, 20, and 81, respectively) [32-35]. No further definitive studies on any of the aforementioned or other eHealth interventions targeting carers for those with psychosis are available to date (of note, a peer-facilitated online educational intervention study conducted in the United Kingdom is yet to be published [36]).

### Coproduction of eHealth Interventions

As eHealth interventions are for autonomous use by the end users, development methodologies commonly integrate users into the process. In the agile methodology commonly used in constructing software, iterative work sequences of technological expert–led *sprints* of developing and delivering working software are based on a brief commissioned by the clients. Through the cycles of set sprints, the developing software is shown to the clients or consumers for feedback, which is used to tune and adjust the software until its completion [37,38]. Although the agile method has been commonplace in the software development field since the 1970s, more recently, the use of participative research methods, which involve key stakeholders and end users at the core of the intervention development process, rather than brokering out the build work with a commission brief, have gained popularity [39-44].

Within mental health research, there is an established tradition of service user–led and survivor-led research, with survivor researchers consistently arguing that, for example, the *closeness* of the researcher to the enquiry increases the validity of study findings [45]. Although such an approach might help ensure that the build process responds closely to the experiences of end users in this study, the literature on, specifically, carer-led research in mental health is extremely limited and seemingly absent in the field of eHealth [46]. In contrast, there is a growing literature documenting participatory design methodologies, including, but not limited to, involving patients, carers, and the public as end users in the core of the research team and research activities, to inform the design and development of interventions [29,41,47]. Contrary to conventional interventions delivered through a face-to-face medium, where the intervention development and delivery are often driven solely by the professionals, consumers of eHealth interventions take on a much more active role [48,49]. In most eHealth interventions, including those that provide guided support from a professional or health care provider, the end users take on responsibility in initiating contact and engaging with the intervention, working through the content, and undertaking self-reported outcome measures online [19,39]. These make it paramount that the end users are involved actively in designing the intervention, not only to make sure the content meets their needs but also to ensure that the way the intervention is delivered keeps them engaged.

Overall, 2 recent systematic reviews on the topic have repeatedly identified the use of participatory research methods in eHealth intervention development as the 1 key factor in determining the acceptability and usability of most eHealth (especially electronic mental health [e-mental health]) interventions [44,50]. Among publicly funded health studies conducted in the United Kingdom, there is evidence to suggest that involving patients and the public in participatory research is positively linked to study success, in terms of recruitment and retention rates [47,51]. It can be argued that the significance of participatory research could only be amplified when it is applied in the eHealth arena where the end users assume much more direct control in using and adhering to the interventions.

### The E-Support for Families and Friends of Individuals Affected by Psychosis Project

**Figure 1:**
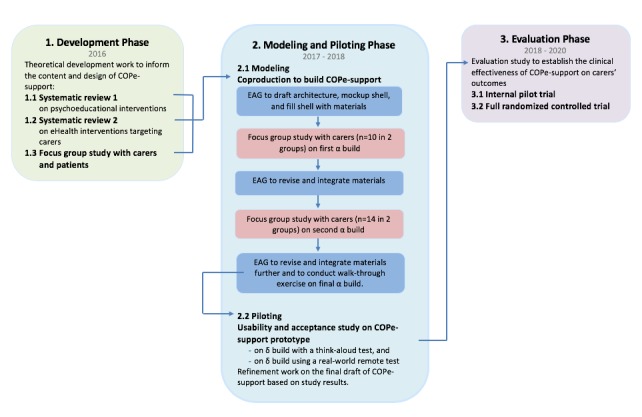
Research methodologies used across the whole EFFIP project focusing on the intervention build and modeling phase. COPe-support: Carers fOr People with Psychosis e-support; EAG: expert advisory group; EFFIP: E-support for Families and Friends of Individuals affected by Psychosis; eHealth: electronic health.

The E-support for Families and Friends of Individuals affected by Psychosis (EFFIP) Project was set up to develop and evaluate an eHealth intervention for carers supporting a relative with psychosis [52]. The overall EFFIP project lasts for 5 years spanning across the theoretical development work to the effectiveness evaluation of the end product on improving carers’ health outcomes based on the Medical Research Council Complex Interventions Framework [53,54]. The design of the overall project is illustrated in Figure 1, focusing on this study.

This study reports the intervention building/modeling phase of the overall EFFIP project (see Figure 1) [53]. Before this phase, we conducted 3 studies in the theoretical development phase. These included 2 systematic reviews and a focus group study exploring research evidence and individuals with psychosis and carers’ ideas and views for the optimal intervention design including essential ingredients, contact hours, and facilitation considerations ([14,19]; also JS et al, unpublished data, 2019). In the build and modeling phase of the study, reported here, we aimed to integrate participatory research with end users alongside core research team activities to coproduce an eHealth intervention prototype to improve flexible access to high-quality psychoeducation and interactive support resources for carers [55,56]. We as a team decided to take this approach, rather than having carers lead the study, in part because of resource constraint and also because we considered that it was important that a range of consumer voices were included across all design decisions. Specific objectives of this study were to apply participatory methods with end users involvement to design and build the intervention, integrate iterative consultations with end users along the rapid prototyping work, report and consider the appropriateness of a participatory approach to eHealth intervention design, and test and refine the final draft of the intervention to get it ready for the usability evaluation, which is reported separately (JS et al, unpublished data, 2019).

## Methods

### Design

**Figure 2:**
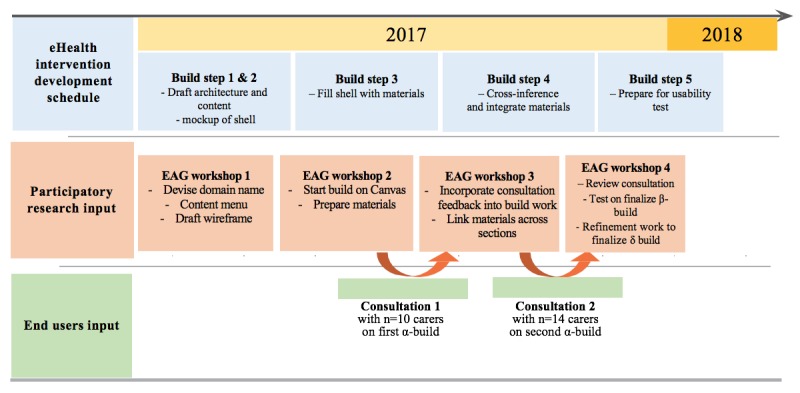
eHealth intervention build process integrating various inputs. EAG: expert advisory group; eHealth: electronic health.

The co-design and build process of the eHealth intervention followed the UK National Institute for Health Research online resource development cycle [57]. This build method was chosen as we were to develop and deliver an eHealth intervention through an existing platform, rather than developing software or the platform ourselves [37,38]. There are 5 build steps illustrated in Figure 2. These are as follows: (1) draft architecture and content, (2) mockup of shell, (3) fill shell with material, (4) cross-inference and integrate materials, and (5) prepare for piloting.

### Coproduction Workshops With an Expert Advisory Group

The co-design and build work were directed by an expert advisory group (EAG) comprising individuals and carers with lived experience of psychosis and professionals working in health, social, or voluntary sectors with the target population. The EAG membership was devised to address the principles of participatory research that value contributions from expertise through experience, and that should never be underrepresented within the bigger whole-team context [47,58]. We recruited EAG members with diverse demographic characteristics and varying degrees of ease and familiarity with digital communications from various clinical and voluntary service provider organizations across South East England. EAG members were paid a goodwill payment for meeting attendance and contributions and were also reimbursed for their travel expenses according to the INVOLVE payment scheme, a UK powerhouse that promotes patient, carers, and public involvement (PPI) in research [59].

The EAG comprised 3 individuals with lived experience of psychosis, 3 family members having different relationships with a loved one affected by psychosis in the family (including a cousin, a sister, and a daughter), 1 clinician who also has personal experience of family caregiving, and 1 voluntary service lead. The EAG worked with the core research team (comprising 2 clinical academics, 1 health services researcher, 2 eLearning experts, and 1 administrator) to lead the build work.

Over the build phase lasting 12 months, 4 EAG workshops (1-4) were held, equivalent to the sprint cycles used in the agile approach. We used the coproduction in mental health improvement work method to organize the workshops [55,56,60]. This approach emphasizes and values the hidden capacity and capability of end users, families, and significant others. At each workshop, all members assumed equal decision-making role, whereas we contributed with our respective strengths and expertise [60,61]. These participatory design workshops (and follow-up work) were mapped to fit the 5-step development process of eHealth products [57], and hence, each had specific aims and expected output, progressing from generating ideas and design to reviewing and refining product from rapid prototyping cycles.

After each participatory design workshop, the knowledge and ideas generated were translated to produce draft hand-sketched plans and wireframes, mockups of Web pages, and source materials for the intervention (eg, videos or textual information). The mockups and output produced between workshops and end users’ feedback obtained from consultations (see below) were then presented at the next workshop, enabling content and broad design ideas to be critically discussed and reviewed and then further developed. Toward the end of the co-design and build work (build steps 4 and 5), the EAG undertook final development work before conducting a walk-through exercise of the online beta-build of the intervention [62-64]. Final revision and refinement work were undertaken in build step 5 to produce the intervention prototype (ie, the delta-build in Figure 2) ready for the real-world usability test.

### Iterative Consultations With Carers

At the interfaces of build steps 3 and 4, we incorporated 2 rounds of iterative consultations with target end users [52]. For the consultations, we recruited carers who had no prior involvement with the EFFIP project to a focus group meeting lasting up to 2 hours. During the first consultation, we showed the carers the offline first alpha-build of the intervention and asked for their feedback on its design and likeability, the flow and readability of the content, and invited them to preempt potential usability issues and identify ways to promote end users’ engagement. The second consultation followed a similar format with another set of carers, with an online second alpha-build of the intervention, which had been further developed by the EAG. The carers’ views were fed back to the rapid prototyping cycles as overseen by the EAG. Figure 2 illustrates the inputs made by the focus groups.

The consultation was approved by the UK National Health Service Research Ethics Committee (REC) process (REC approval reference number: 16/LO/1300) and Health Research Authority (HRA IRAS project ID: 210571). Carers aged 18 years or older and who provided unpaid support and care for a loved one affected by psychosis were recruited from 3 mental health trusts in South London and Berkshire, South East England. Recruitment strategies for carers included the posting of study flyers at clinical areas and informing the carers support workers and mental health professionals at each trust to disseminate the study information to carers.

### Analysis Strategies

All the workshops and consultation meetings were digitally recorded and transcribed verbatim. We took photos of the sketches produced at the workshops, on which the wireframes were developed later. The qualitative data were analyzed using the thematic analysis method [65,66], suiting the social constructionist and realist paradigms. We used an inductive approach to identify themes from the data without trying to fit it into a preexisting coding frame but grounded our understanding on the EAG members’ and carers’ perception and experiences of the evolving drafts of the intervention. As the study focused on designing and reviewing the build of the intervention, the data were analyzed descriptively to generate themes concerned broadly with the design and content of the intervention, general look and feel, usability factors, privacy and security, and ways to enhance engagement and usefulness.

Throughout the study, to ensure the rapid prototyping and build of the intervention was grounded in the data, the participatory design workshops, focus group consultation, and data analysis were performed in parallel with one another. This ensured timely and robust feedback to inform the evolving drafts of the intervention (see Figure 2).

## Results

### Expert Advisory Group Members and Consultation Group Participants

The EAG comprised 8 members, whereas the core team had 6 members. Over 1 year during which 4 participatory workshops were held, all EAG members and core team members attended all the workshops and selected follow-up work to create the materials as indicated.

During each round of consultations, 2 meetings involving 5 to 8 carers were held (see Figures 1 and 2). In total, 24 carers participated in the iterative consultations along the evolving build process: 10 on the first alpha-build, and 14 on the second alpha version. The participants comprised 10 men and 14 women. The range of the carers’ ages was 22 to 83 years (mean 59 [standard deviation, SD 12.7]) with the median age being 61 years. Half of the carers had retired (n=9) or stayed at home being a full-time carer (n=3). The other half were in gainful employment: 5 working full time, 6 working part time, and 1 actively seeking employment. Most of the carers were a parent (19/24, 79%), and there were 2 spouses (8%, 2/24), 2 close friends (8%, 2/24), and 1 adult child (4%, 1/24). Just more than one-third of the carers (38%, 9/24) lived with their cared-for person at the time of the study. The gender mix of the cared-for persons was similar to that of the carers; 11 were male (46%, 11/24), and 13 were female (54%, 13/24). The ages of the cared-for persons ranged from 17 to 61 years (mean 35.3 [SD 15.8], median=32). In terms of diagnosis, half of the participants reported that their cared-for persons (50%, 12/24) had a diagnosis of psychosis, 9 were diagnosed with a schizophreniform disorder (45%, 9/24), and 3 had type 1 bipolar disorder (13%, 3/24). As reported by the participants, the cared-for persons had been unwell for less than 1 year to the longest for 36 years (mean 10.6 [SD=10]; median=6). The demographic characteristics and caring situation of the participants and their cared-for persons are summarized in Table 1.

**Table 1:** Summary of focus group consultation participant characteristics and caring situation.

Characteristics		Carer participants (n=24)	Their cared-for person (n=24)
**Age**			
	Mean (SD^a^)	59 (12.7)	35.3 (15.8)
	Median (range)	61 (22-83)	32 (17-61)
**Sex**			
	Male, n (%)	10 (41)	11 (46)
**Ethnicity, n (%)**			
	White	18 (75)	—^b^
	Black	1 (4)	—
	Asian	2 (8)	—
	Other	3 (13)	—
**Work, n (%)**			
	Full-time work	5 (21)	—
	Part-time work	6 (25)	—
	Retired	9 (38)	—
	Not working	1 (4)	—
	Looking after home/family	3 (13)	—
**Marital status, n (%)**			
	Single	1 (4)	—
	Married/cohabiting	17 (71)	—
	Other	6 (25)	—
**Relationship with the cared-for person, n (%)**			
	Parent	19 (79)	—
	Spouse/partner	2 (8)	—
	Close friends	2 (8)	—
	Child	1 (4)	—
**Accommodation arrangement of carer, n (%)**			
	Living with cared-for person	9 (37)	—
	Not living with cared-for person	15 (63)	—
**Diagnosis of cared-for person, n (%)**			
	Psychosis	—	12 (50)
	Schizophreniform disorder	—	9 (37)
	Type 1 bipolar disorder	—	3 (13)

^a^SD: standard deviation.

^b^Not applicable.

### Expert Advisory Group Coproduction Workshops and Iterative Consultations

#### Expert Advisory Group Workshop 1

The inaugural workshop was set to design the study website and the online intervention, including their (domain) name, content, and look. The EAG was provided with results obtained from prior studies conducted in the theoretical development phase of the EFFIP project ([14,19]; also JS et al, unpublished data, 2019) to aid their design decision.

**Figure 3:**
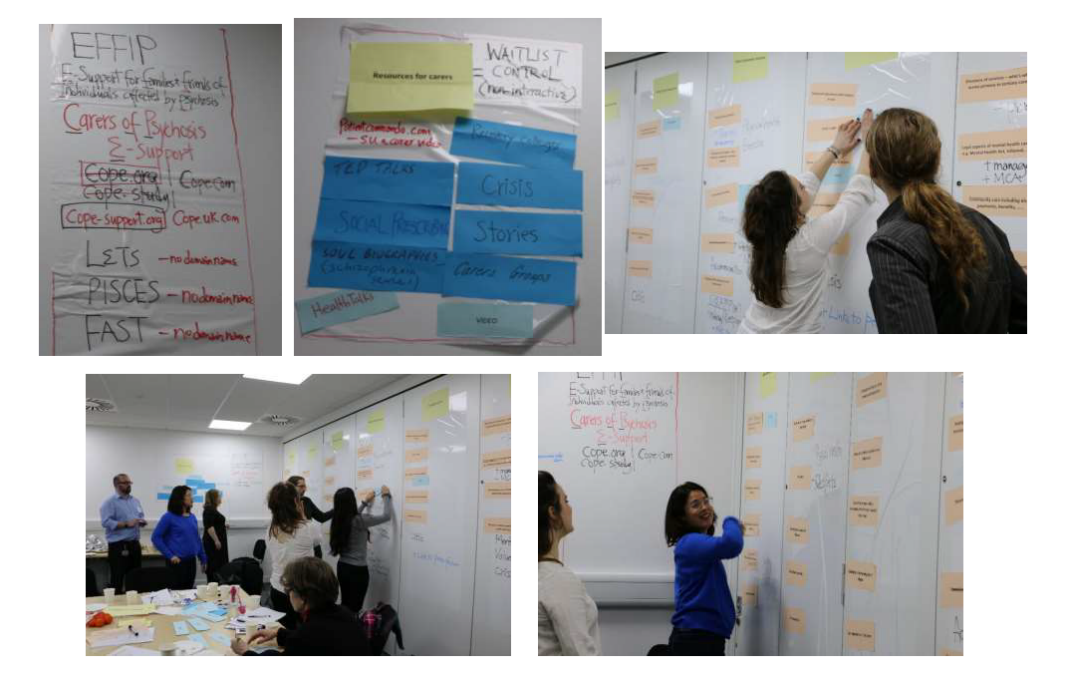
Samples of early designs and sketches regarding the project website and the eHealth intervention devised by the EAG. COPe: Carers fOr People with Psychosis e-support; EAG: expert advisory group.

Following an extended brainstorming exercise, we decided to call the eHealth intervention *COPe-support*, an acronym of Carers fOr People with Psychosis e-support. We subsequently secured the domain name of *cope-support.org* and hosted our project website on the World Wide Web. With carers as end users in mind, we set out to design a clean and user-friendly website giving information about the project and its research program. The EAG believed that the testimonials from other carers who helped develop the intervention would add credibility to the intervention; hence, these testimonials and photos of the EAG working together were added to the website (see Figure 3).

The EAG reviewed the essential ingredients as identified by our earlier development work (JS et al, unpublished data, 2019) to produce the *draft architecture and mockup of shell* (build step 1). We used nondigital design tools such as magic sheets, post-it notes, index-cards with content items written on them, papers, and pens to encourage a creative design atmosphere involving all members, regardless of their competency level of information and communication technology. EAG members did hand-drawn sketches of Web pages and organized the content and ingredients in a structure that they saw fit.

The EAG decided the master plan for the content and key functions of COPe-support. The intervention comprised 10 sections of psychoeducational materials, communication and problem-solving knowledge and skills, reflective exercises, and discussion points. There were 2 interactive discussion forums: one with a panel of expert members comprising professionals and experts through experience and another one with carers as peers. Figure 3 provides samples of early designs and sketches regarding the project website and the intervention.

#### Expert Advisory Group Workshop 2

The second workshop was held 6 weeks later. For hosting and running COPe-support, our eLearning experts (LW and AS) identified a virtual learning environment (VLE) called Canvas [67]. The COPe-support intervention, accessible via the Canvas VLE, was designed to work on desktop (or laptop) Web browsers, as well as smartphones or tablets through a Canvas app.

Building on the framework and functions of Canvas, the EAG members worked together to *build shell with materials* at build step 2 [57]. We structured all the content items onto 1 platform hosting 12 sections. These include the following:

- Two modules on psychosis, common symptoms, and comorbid problems and evidence-based treatment for psychosis;
- Two modules on caring strategies for common symptoms and problems (eg, supporting your loved one with paranoid beliefs) and on ways to promote recovery;
- Two modules on wider social and service issues related to psychosis including ways to deal with stigma and discrimination and navigating the health and social care systems;
- Two modules focusing on well-being–promotion strategies for carers themselves;
- A virtual discussion forum and blog space for carers to share experiences and discuss commonly encountered issues;
- An *Ask the Experts* forum where participants can post questions to an expert panel comprising health and social care professionals and campaigners;
- A *Further Resources* section with supplementary Web links to relevant external resources; and
- A support page where carers can get in contact with the online facilitator for technical or emotional support directly.

During this workshop, the EAG members also discussed the best ways to present the different elements and topics. We devised a detailed work plan to source or produce the materials in various formats, ranging from textual documents to making videos with experts speaking on the specific topics and Weblinks to external sites (with explicit agreement sought).

**Figure 4:**
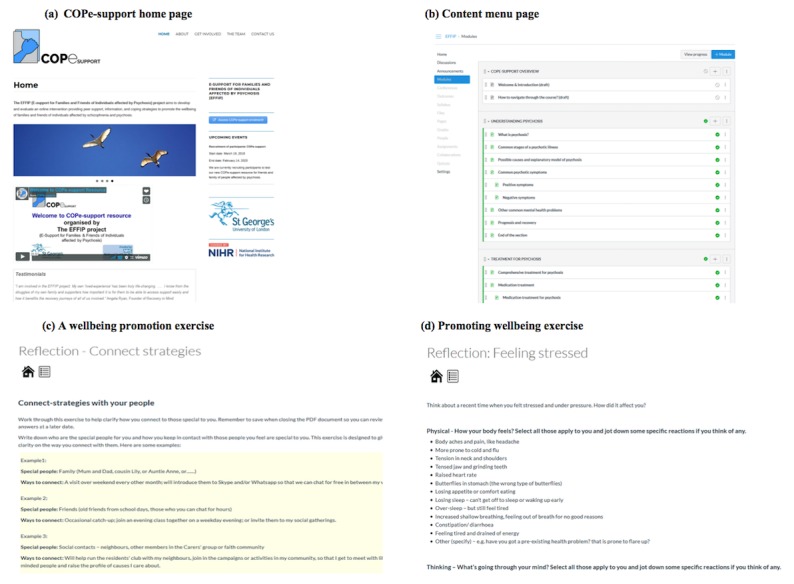
Screenshots of the first alpha-build of COPe-support. (a) COPe-support home page; (b) content menu page; (c) a well-being promotion exercise; (d) promoting well-being exercise. COPe: Carers fOr People with Psychosis e-support.

The following 2 months after these workshops saw the EAG producing the materials and the sketch design of the intervention being turned into wireframes with materials developed filling the shell—the build step 3 [57]. Figure 4 provides screenshots of the first alpha build of COPe-support.

#### Consultation With End Users on the First Alpha-Build

Once the first alpha-build of COPe-support (including the home page where the intervention menu and content list sat and mockups of 3 modules and the 2 forums) was prepared, we ran the first consultation showing the carers its offline version to seek their feedback.

Feedback from carers indicated that the general presentation and the content of COPe-support were well received. However, carers found the linear program of sections and elements too prescriptive (see Figure 4), whereas they envisaged that carers as end users would prefer flexibility in choosing relevant content suiting their own caring situation. Although carers rated positively the differing elements including psychoeducational information, reflective exercises, and practice guides, which encourage the integration of skills learnt into their day-to-day life, they found the default terminology used by the Canvas VLE alienating. Some examples included the terms *quizzes* and *grades*. The findings obtained from the focus groups were fed back to the rapid prototyping cycle overseen by the EAG.

#### Expert Advisory Group Workshop 3

**Figure 5:**
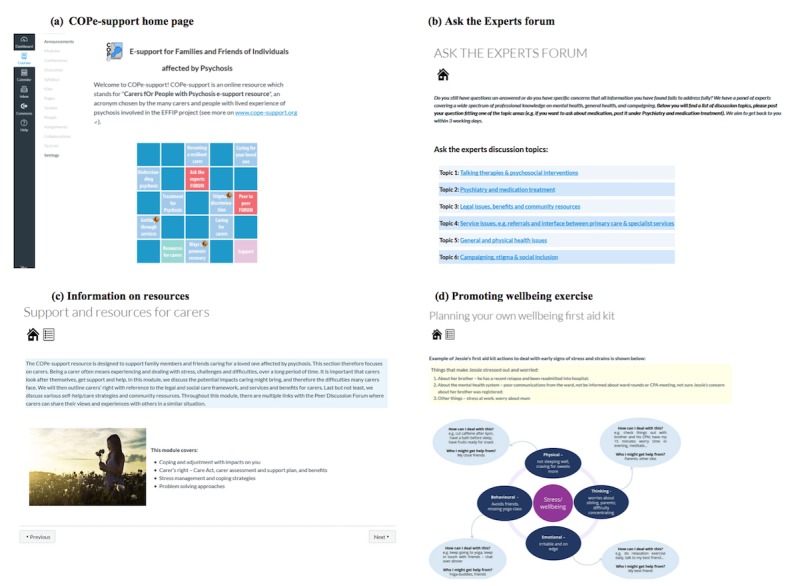
Screenshots of the second alpha-build of COPe-support. (a) COPe-support home page; (b) ask the experts forum; (c) information on resources; (d) promoting well-being exercise. COPe: Carers fOr People with Psychosis e-support.

Although the rapid prototyping build work progressed onto build step 4—*cross-inferencing and integrating materials*, the consultation findings on the initial alpha-build of COPe-support were reviewed by the EAG at this workshop. The original linear program menu design was changed to a grid-based visualization, which implied no order for its content yet still provided the functionality to link to the relevant areas. The 3 key elements, that is, psychoeducational information, interactive forums, and further resources, were color-coded in the content menu. Explicit guidance notes were added to encourage participants to pick and choose the content relevant to their needs. We changed the terminology used by the Canvas VLE, which was originally designed for eLearning students, as much as possible to suit the carer population. These included labeling all the exercises and prompts for reflection and integration as *reflection* and leaving out *submission or grading* and *sharing* of reflections through peer-to-peer forum as some participants might perceive that as a source of unintended pressure. In both forums, we added ground rules and notes to explain the privacy and security measures and provided examples in writing posts or questions without giving personally identifiable data away. We also developed further sections using these design principles in producing the second alpha-build of COPe-support. Figure 5 provides screenshots of the second alpha-build of the intervention.

#### Consultation With End Users on the Second Alpha-Build

A second round of focus group consultation was subsequently organized when we showed the carers an online version of the second alpha-build of COPe-support (see Figure 5). Carers’ feedback for this was positive in general. They found the home page welcoming and particularly appreciated the content menu design, which they found inviting and intuitive to use. The explicit display of the ground rules and guidance notes to ascertain privacy and security in the home page and again in forums was identified by carers as enhancing their sense of trust and ease of use. The layout of pages focusing on psychoeducational information given was well evaluated with the use of pictures and graphics to intercept the text. We asked carers to review the 2 forums and the discussion topics during the consultation. Most found that the topics gave some structures for the participants to focus their posts and felt confident and comfortable to use the forums with the guidance notes and the anonymous participation arrangement. Carers identified that there was a need for clearer instruction in how the participants can contact the COPe-support facilitator directly for support if necessary. They liked the cognitive behavioral–oriented reflections and exercises that promote self-care but would like more instruction on downloading the materials to allow practicing in their own time.

#### Expert Advisory Group Workshop 4

In the 2 months between the last consultation and the fourth coproduction workshop, further rapid prototyping work was carried out to address the focus group findings.

Workshop 4 concluded the build step 4— *cross-inference and integrating materials* —of the coproduction work and preceded the final build step 5— *ready for usability test*. During this workshop, the EAG members conducted a walk-through exercise as a group on the beta-build of COPe-support [62-64]. Through the walk-through exercise starting from *login* to *log-off*, the EAG members navigated through every section and tried out every function of COPe-support from an end user’s perspective. A number of minor usability issues related to navigation and use of various functions were identified (eg, how to raise a new post?). We took note of these findings and carried out further refinement accordingly. We also captured the EAG members’ experience and feedback expressed through the exercise to inform the production of a navigation video (and a written navigation guide in parallel).

We discussed and confirmed the privacy and data security measures based on the exercise and the data output from it. In addition to the anonymous participation mechanism through the use of pseudonyms by all enrolled carers, the EAG considered it useful to state explicitly the monitoring, moderation, and facilitation provided by a qualified mental health professional (JS) daily during the week. We believed that it helped create and maintain a safe online environment as well as enhance the credibility of the intervention. The facilitator also posts weekly updates on COPe-support online forum to all carers with an aim to keep them engaged.

These considerations and final refinement work were undertaken in the final build step 5— *preparing for usability test* —with the delta-build of COPe-support developed by the end of the build and modeling phase.

## Discussion

### Principal Findings

Our study used an innovative approach to develop a Web-based intervention for psychosis carers. COPe-support provides psychoeducation and emotional support using health care professional contribution and peer support [52]. The participatory research design method ensured that carers as end users were fully involved in all phases of the design and build process [47,60,68,69]. We believe that carers, together with other key stakeholders such as individuals with lived experience of psychosis and health care professionals, provided some insightful foresight of target end users’ expectations and usage pattern for the intervention. These were addressed and taken on board in the build process to optimize the matching of end users’ expectations and needs and the final product design and delivery. Furthermore, these end users’ inputs helped the core team, especially the eLearning experts, to understand how best to manipulate the technological remits as afforded by our chosen software to enhance the relevance and suitability of the intervention for the end users ([63,64]; also JS et al, unpublished data, 2019).

Our study combined 2 distinct participatory research coproduction methodologies with end users—the participatory research workshops to design and build the intervention and consultation meetings to provide feedback on drafts along the rapid prototyping cycles—in parallel and interacting with one another [29,52,58]. Furthermore, 2 different groups of end users and stakeholders were involved in the different methods and activities (carers with no involvement with the project joined the consultation meetings on initial and second alpha-builds, alongside an established set of EAG members). As we embedded the participatory research elements within a structured eHealth build model originally developed for e-mental health interventions [57], the repeated development cycle borrowed from the agile method enhanced the continuous generation of new ideas and feedback being inputted into the coproduction build process. Our approach integrating PPI into the agile build process, enabled end users to have hands-on involvement in producing and revising the developing intervention drafts; their experiential insights were not lost through interpretation by external technological experts which, we hope, the insights gained likely to increase end users’ engagement with the intervention [47,60]. We would argue that our participatory approach was better suited than a carer-led method, as we were able to integrate a full range of stakeholders, including eLearning experts, clinicians, and researchers into the build process alongside carers.

### Limitations

The participatory design and build work described in this paper have produced a final prototype of COPe-support. This study has produced a tangible output, that is, an eHealth intervention meeting the required and desired expectations of both EAG members and the carer participants who contributed to the iterative consultation alongside the prototype development. However, our results are descriptive in nature and limited in establishing the usability or acceptability of the intervention, and our sample of carers was limited by size and representativeness. The success of our approach will ultimately be tested in experimental evaluation of the intervention itself (see below).

### Implications and Future Directions

This study illustrates the importance of coproduction of COPe-support, an eHealth intervention, which is designed to be used by carers autonomously. Another important contribution this study made to research in the field is the documentation of a rigorous and innovative build process, which combined intervention development method and participatory research methodologies throughout its life cycle [69]. Across the spectrum of eHealth intervention build methods, we recognize our participatory research method being in the middle ground of the 2 polar approaches: agile or technologist-driven and carer-led. Our approach establishes itself as a third way adopting the agile process and principles while integrating PPI with the technological and research core team conducting the build work as directed by the EAG.

Following this study with the prototype ready for feasibility and usability testing, a usability study of the final delta-build of COPe-support has been completed with both a remote usability trial and a think-aloud study (JS et al, unpublished data, 2019). The results were promising and provided further feedback from end users, in terms of facilitation and delivery strategies (JS et al, unpublished data, 2019). Following further refinement work as informed by the usability study (JS et al, unpublished data, 2019), COPe-support is currently being tested for its effectiveness in supporting carers through an online randomized controlled trial (RCT) [54]. The trial will also test levels of carers’ engagement both with the intervention and the study and for any correlation of engagement or adherence with the effects on carers’ health outcomes. These investigations will therefore enable us to consider, in the future, if our participatory research approach has been successful.

To the best of our knowledge, COPe-support is one of the few comprehensive Web-based interventions targeting carers for psychosis patients. With the benefits of access, facilitation, and delivery completely through the internet, our product has the potential to provide an evidence-based psychoeducational intervention with its key ingredients [14,19]. These include providing health outcomes monitoring, psychoeducational information, and real-time interaction with health care professionals and other carers as peers. We expect the internet delivery will overcome some of the implementation and access barriers from both the service providers’ and carers’ perspective [19]. Dependent on the trial outcomes, we have considered several areas for further development and innovation in the future. These range from investigating further implementation strategies to optimize large-scale rolling out of COPe-support upon positive trial results to examining and incorporating additional health behavioral change techniques or methods used in eHealth interventions to enhance engagement and effects upon unclear effectiveness results [70,71]. Participatory research methods and principles will, no doubt, be an important approach integrated within such work.

### Conclusions

We integrated participatory research methodologies with a structured eHealth intervention development process to develop COPe-support through this study. COPe-support is one of the few eHealth interventions dedicated for family carers of individuals affected by psychosis. It provides information and psychosocial support for carers through the internet, promoting flexible access and individualized choice. Following usability evaluation of the intervention prototype, we are currently undertaking an online RCT to evaluate its effectiveness in promoting carers’ health outcomes.

## Abbreviations

COPe-support: Carers fOr People with Psychosis e-support
EAG: expert advisory group
EFFIP: E-support for Families and Friends of Individuals affected by Psychosis
eHealth: electronic health
e-mental health: electronic mental health
NIHR: National Institute for Health Research
PPI: patient, carers, and public involvement
RCT: randomized controlled trial
REC: Research Ethics Committee
VLE: virtual learning environment
